# Influence of LDL cholesterol and Lp(a) on monocytes and macrophages in atherosclerosis

**DOI:** 10.11613/BM.2025.030503

**Published:** 2025-10-15

**Authors:** Sabina Ugovšek, Jernej Jeras, Miran Šebeštjen, Janja Zupan

**Affiliations:** 1 Department of Cardiology, University Medical Centre Ljubljana, Ljubljana, Slovenia; 2 Faculty of Medicine, University of Ljubljana, Ljubljana, Slovenia; 3 Community Health Centre Ivančna Gorica, Ivančna Gorica, Slovenia; 4 Department of Vascular Diseases, University Medical Centre Ljubljana, Ljubljana, Slovenia; 5 Faculty of Pharmacy, University of Ljubljana, Ljubljana, Slovenia

**Keywords:** atherosclerosis, monocytes, macrophages, lipoprotein(a), LDL cholesterol

## Abstract

Atherosclerosis is an active interaction between lipoproteins and inflammatory cells. Monocytes and macrophages are the most important immune cells involved in the process of atherosclerosis. They interact with atherogenic lipoproteins, in particular low density lipoprotein (LDL) cholesterol and lipoprotein(a) (Lp(a)). The increased concentration of the LDL cholesterol and Lp(a) accelerates the polarization of monocytes and macrophages toward proinflammatory phenotype and the formation of the foam cells. These cells then release large quantities of inflammatory cytokines that stimulate the oxidation of atherogenic lipoproteins that are even more atherogenic and contribute to the formation of foam cells and the secretion of the pro-inflammatory cytokines, thus creating a vicious circle. Surface marker C-C chemokine receptor type 2, expressed on monocytes/macrophages, enables their adhesion and migration into the subendothelial layer. The rupture of the atherosclerotic plaque on one hand, and the ability of the oxidized LDL cholesterol and Lp(a) to trigger arterial thrombosis by different mechanisms on the other hand, result in acute cardiovascular event. Here, we summarize the role of the monocytes and macrophages in atherosclerosis and explore the influence of LDL cholesterol and Lp(a) on monocytes and macrophages during the entire process of atherosclerosis, from its initiation to progression.

## Graphical abstract

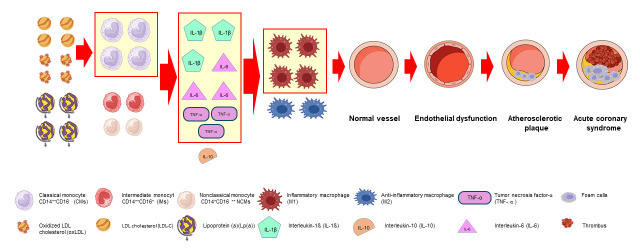

## Highlights

• Inflammatory cytokines stimulate the oxidation of atherogenic lipoproteins

• Dyslipidemia triggers transformation of monocytes toward proinflammatory phenotype

• Monocytes and macrophages participate in plaque formation and post rupture events

• Surface marker C-C chemokine receptor type 2 enables monocytes migration into the subendothelial layer

## Introduction

Atherosclerosis with its clinical consequences, especially ischemic heart disease, cerebrovascular diseases and peripheral arterial disease, is one of the most common causes of morbidity and mortality (*1*). For more than three decades atherosclerosis has been considered as a passive process of lipids deposition in the vascular wall with the consequent reduction of the vascular lumen. The process of the formation of the atherosclerotic lesions begins with impaired function of the endothelium and continues with morphological changes such as the formation of fatty streaks, followed by the development of atherosclerotic plaques and their rupture resulting in acute cardiovascular event (*2*). Since inflammation is actively involved in all phases of the atherosclerotic process, atherosclerosis can be considered as a low-grade lifelong active inflammatory process (*3*). Monocytes are the most important cells of the immune system involved in the process of atherosclerosis. The link between monocytes and atherosclerosis is unequivocal, and an increased number of monocytes is causally related to the complications of atherosclerosis (*4*). Mobilisation of monocytes into the subintimal space starts as early as in childhood and fatty streaks can be present already in adolescents and young adults (*5*). After infiltration into the endothelium, monocytes differentiate into macrophages, which can be either pro-inflammatory (M1) or anti-inflammatory (M2) (*6*). M1 macrophages, by secreting pro-inflammatory cytokines such as interleukin (IL)-1β, IL-6 and tumor necrosis factor-α (TNF-α), contribute to the accelerated growth of the atherosclerotic plaque and, in the advanced stage, to its greater instability and possibility of rupture, resulting in an acute cardiovascular event. On the other hand, M2 macrophages, by secreting anti-inflammatory factors such as IL-1 receptor (IL-1R) antagonist, IL-10 and collagen, contribute to a slower progression of atherosclerosis and greater stability of the atherosclerotic plaque, thereby reducing the possibility of its rupture and occurrence of an acute cardiovascular event (*7*). However, the role of monocytes and macrophages in atherosclerosis is not solely limited to the growth and stability of the atherosclerotic plaque. Monocytes and macrophages also participate in the regulation of the coagulation-fibrinolytic system, hence their impact is also important in the case of the eventual rupture of the atherosclerotic plaque.

Macrophages and smooth muscle cells within the atherosclerotic plaque oversecrete tissue factor (TF) into the blood flow. Tissue factor initiates activation of the extrinsic coagulation pathway, which leads to thrombus formation and fibrin deposition in the intima (*8*). Monocytes are also capable of secreting plasminogen activator inhibitor (PAI) 1 and 2, as well as tissue type plasminogen activator (t-PA) and urokinase type plasminogen activator (u-PA), which suggests that monocytes can control the expression of plasmin on their surface (*9*). In this way, monocytes and macrophages influence both, the tendency of the atherosclerotic plaque to rupture, as well as the eventual thrombotic events in the ruptured atherosclerotic plaque. By secreting thrombogenic and/or fibrinolytic activators and/or inhibitors, they can significantly affect the occurrence of acute cardiovascular events.

Dyslipidemia, in particular increased concentrations of low-density lipoprotein (LDL) cholesterol and also lipoprotein (a) (Lp(a)), which is a lipid risk factor that, independently of LDL cholesterol concentration, increases the risk of future cardiovascular events, is influenced by monocytes/macrophages and thus the atherosclerotic process (*10*, *11*).

**Figure 1:**
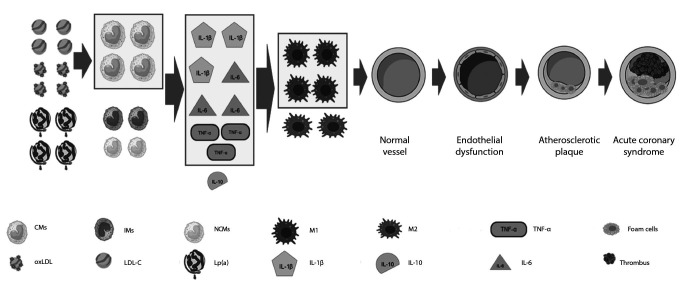
Increased concentrations of lipoprotein (a) (Lp(a)), LDL cholesterol (LDL-C) and consequently oxidized LDL cholesterol (ox-LDL) trigger increased trans-formation of monocytes to a more proatherogenic subtype (marked with yellow square). These processes accelerate the synthesis of the proinflammatory cytokines (marked with yellow square) and reduce the synthesis of the antiinflammatory cytokines causing transformation of the macrophages into a proinflammatory subtype (marked with yellow square). This leads to a faster formation of the unstable atherosclerotic plaque resulting in an acute cardiovascular event. CMs - classical monocyte CD14**CD16*. IMs - intermediate monocyte CD14**CD16*. NCMs - nonclassical monocyte CD14**CD16*. M1 - inflammatory macrophage. M2 - anti-inflammatory macrophage. TNF-α - tumor necrosis factor-α. oxLDL - oxidized LDL cholesterol. LDL-C – low density lipoprotein cholesterol. Lp(a) - lipoprotein(a). IL-1β - interleukin-1β. IL-10 - interleukin-10. IL-6 - interleukin-6. The figure was created with BioRender.com.

In the current review, we aimed to shed light on the influence of LDL cholesterol and Lp(a) on monocytes and macrophages during the entire course of the atherosclerotic process. We summarize the influence of monocytes and macrophages on the endothelial function, the formation of the atherosclerotic plaque and the events following its rupture (Figure 1). Interestingly, there is evidence that monocytes and macrophages play an important role in the composition of the formed plaque which forecasts the tendency for its rupture. Moreover, these cells also participate in post rupture events and moderate the outcomes which can vary from a slow progression of the atherosclerotic narrowing to an acute cardiovascular event.

## Monocytes and macrophages

Monocytes constitute 3-8% of all leukocytes in peripheral blood. They represent the main part of the innate immune system, which is responsible for defence against external infections such as bacterial, viral and fungal, mainly with the help of phagocytosis. However, for the development of atherosclerosis their participation in the endogenous inflammatory processes is more important (*12*). The link between monocytes and atherosclerosis is unequivocal, as an increased number of monocytes is causally related to the complications of atherosclerosis (*4*). Nevertheless, monocytes represent a very heterogeneous cell population that play different roles in the process of atherosclerotic lesion formation. The first attempts to classify monocytes were based only on morphological criteria, mainly their size and volume, thus only two groups of small and large monocytes were identified (*13*). The development of new technologies such as flow cytometry and fluorescence-activated cell sorting enabled more accurate classification, mainly by identifying the expression of two cluster of differentiation (CD) surface markers, CD14, which is a lipopolysaccharide (LPS) receptor, and CD16, which is an FcγIII receptor (*14*). Based on these two markers, the monocytes are divided into CD14^++^CD16^-^ (classical monocytes (CMs)), CD14^++^CD16^+^ (intermediate monocytes (IMs)) and CD14^+^CD16^++^ (non-classical monocytes (NCMs)) (*15*). Several attempts to include additional surface markers to the conventional CD14- and CD16-based panel indicate large heterogeneity of the monocyte population (*16*-*18*). However, due to the use of different methods for determining these markers and different combinations of these markers, the identification of the new subpopulations of monocytes remains challenging. Of course, the question persists as to whether these newly identified subpopulations truly represent new monocyte phenotypes, or are solely minimal variations of the existing subpopulations (*19*).

### Classical monocytes

The CMs are inflammatory cells that express high levels of C-C chemokine receptor type 2 (CCR2) on their surface and account for more than 90% of the monocytes (*20*). These cells secrete large amounts of inflammatory cytokines such as IL-1, IL-12 and TNF-α following their release to the sites of active inflammation under the influence of various factors from both, bone marrow and splenic reservoirs. In addition to their propensity to penetrate the endothelial barrier and accumulate in the subendothelial space, CMs possess a very pronounced phagocytic activity (*21*). The lifespan of CMs is very short (approximately 1 day), and only 1% of all CMs are further transformed into IMs (*22*).

### Intermediate monocytes

Similar to CMs, IMs also express high levels of CCR2 on their surface and exhibit a very high phagocytic capacity, which is particularly important in eliminating apoptotic cells after myocardium necrosis, most often in the context of ischemic heart disease (*21*). During development and progression of the atherosclerosis, IMs present a significant source of reactive oxygen species (ROS) and mediators of inflammation, such as TNF-α and IL-1β (*23*). Most of the evidence shows that IMs in particular are directly involved in the progression of the atherosclerotic process, as well as in the acute cardiovascular events. The proportion of IMs proved to be an independent predictive factor for future cardiovascular events in a group of 951 patients with and without previously known cardiovascular disease referred for coronary angiography (*24*). Similarly, the proportion of IMs was identified as an independent predictive factor for the first future cardiovascular event in a group of 229 patients with known stable coronary disease, independently of other risk factors including pro-inflammatory cytokines produced in all three subtypes of monocytes (*25*). Conversely, CMs were found to be an independent predictor of future cardiovascular events in 700 patients without clinically evident cardiovascular disease (*26*). However, the latter study was performed only in patients without clinically identifiable cardiovascular disease. Moreover, the analysis was performed on samples frozen for up to 15 years, whereas the other two studies analysed monocytes in fresh samples. Hence, the controversy might arise from these two obvious differences between the three studies.

The association between IMs and risk factors for cardiovascular disease, particularly dyslipidemia is also in favour of IMs. The proportion of IMs was significantly higher in patients with increased Lp(a) compared to those with normal Lp(a) values as shown in the study on patients with stable coronary disease (*27*). On the other hand, the proportions of CMs and NCMs were the same in both patients’ groups (*27*). However, only the proportion of IMs, but not the other two monocyte subtypes, was associated with the oxidized phospholipids (OxPLs)/apolipoprotein B-100 ratio in the group of patients with elevated Lp(a) values. Oxidized phospholipids are a well-recognized proinflammatory marker that also activate monocytes (*28*).

### Nonclassical monocytes

After approximately 4 days, all the IMs in the circulation turn into NCMs and remain in the bloodstream for up to 7 days (*22*). In contrast to CMs and IMs that express CCR2 on their surface, NCMs express CX3C motif chemokine receptor 1 (CX3CR1). The latter is primarily expressed on circulating monocytes, tissue macrophages, and tissue dendritic cell populations, but also on T cells and natural killer cell subsets (*29*). Nonclassical monocytes actively and continuously patrol the luminal side of the vascular endothelium both, at steady state and during inflammation, and also mediate the removal of damaged endothelial cells from the vasculature. In addition, they also secrete large amounts of anti-inflammatory and wound healing factors such as IL-1R antagonist, IL10 receptor, apolipoproteins apoA and apoE, and C-X-C motif chemokine ligand 16 (CXCL16) (*30*). Nonclassical monocytes scavenge and accumulate lipoproteins, including the most proatherogenic oxidized lipoproteins, from the vessel wall. Even more important, their number increases in response to the increased concentration of cardiovascular risk factors, in particular the OxPLs (*31*). In the absence of NCMs, the number of pro-inflammatory monocytes and subsequently the macrophages increase, which leads to faster progression of the atherosclerosis (*32*).

### Macrophages

After entering the vessel wall, monocytes can differentiate into macrophages. Depending on the balance between their pro-inflammatory or anti-inflammatory properties, macrophages are classified as M1 macrophages or M2 macrophages, respectively. Even though such a classification seems to be oversimplified, it helps us to define the characteristics of the macrophages, which can vary continuously between both endpoints, *i.e.* the M1 and M2 phenotypes. Having said that the phenotype of the macrophages is also highly dependent on the influences from their microenvironment. Therefore, the macrophages are most commonly referred as M1- or M2-like macrophages, more accurately reflecting their heterogeneity (*33*). The M1 macrophages substantially express CD86 and CD80 along with major histocompatibility complex class II (MHC II) on their surface, which enables the antigen presentation and subsequent activation of the T cells. Interferon (INF)-γ, TNF-α and LPS are the most common activators that direct the polarization of the naïve (M0) to M1-like macrophages. Upon their polarization, M1-like macrophages secrete larger amounts of TNF-α, IL-1β, IL-6, IL-12, IL-23 and ROS (*34*). On the other hand, the most common activators of M2-like macrophages are IL-4, IL-10 and IL-13. The M2-like macrophages secrete larger amounts of IL-1R antagonist, IL-10, transforming growth factor β (TGF-β), as well as other cytokines and chemokines (*35*).

## Monocytes and macrophages in the initiation of atherosclerosis

Endothelial dysfunction is an early indicator of atherosclerosis, a systemic vascular disease associated with reduced synthesis of nitric oxide, which participates in arterial dilation. Endothelial dysfunction is a merit indicator of the progression of the atherosclerosis and the risk of coronary events (*36*). Impaired endothelial function is not only present in patients with evident cardiovascular disease, it also shows in apparently healthy individuals with existing risk factors (*37*, *38*). Inflammatory cells, including monocytes and monocyte-derived macrophages, are extremely important for the initiation of the endothelial dysfunction. With the help of the cell-surface proteins, *i.e.* selectins, monocytes bind weakly and reversibly to cytokine-activated endothelial cells (ECs). The ECs are activated with the inflammatory cytokines, in particularly with TNF-α, IL-1β and IL-6 (*39*). This activation induces the expression of the adhesion molecules such as E- and P-selectin, intercellular adhesion molecule 1 (ICAM-1) and vascular cell-adhesion molecule 1 (VCAM-1) that participate in monocyte migration (*40*). The adhesion of monocytes and monocyte-derived macrophages is followed by their polarization to one of the subtypes of monocytes or monocyte-derived macrophages and their migration into the subendothelial layer (*41*).

One of the most important risk factors, which decisively affects the direction of the polarization of the monocytes and macrophages, is hypercholesterolemia, in particular an increased concentration of the LDL cholesterol. Escate *et al.* found that atherogenic concentrations of the LDL cholesterol significantly shorten the time needed for differentiation of the monocytes adhered to ECs into macrophage-like cells (*11*). This influences both, the morphology of the monocytes and the level of their expression of surface marker CD14. The expression of CD14 was shown to be significantly reduced when monocytes are exposed to atherogenic concentrations of LDL cholesterol in comparison with placebo. An increased concentration of LDL cholesterol does not only accelerate the polarization of monocytes towards proinflammatory or proatherogenic phenotype, but also increases the production of monocytes in the bone marrow (*42*). Interestingly, the concentration of LDL cholesterol was not related to the number of leukocytes, but only to the number of monocytes and granulocytes, independently of the concentration of C-reactive protein (CRP) (*42*). Compared to the patients with normal LDL cholesterol concentrations, Bekkering *et al.* showed higher expression of CCR2 on monocytes from untreated familiar hypercholesterolemia (FH) patients that persists even after treatment with statins and proprotein convertase subtilisin/kexin type 9 (PCSK9) inhibitors and ezetimibe if required (*43*). The reason for this is persistent hyper responsiveness of the circulating monocytes or so-called “trained immunity” (*43*). Given that monocytes have a life span of several hours to several days, while the treatment in this study lasted for 12 weeks, the authors suspect the reprogramming of the progenitor cells in the bone marrow under the influence of an increased concentration of the LDL cholesterol (*43*). Progenitor cells in the bone marrow present the source of the new monocytes in the peripheral circulation. This was further confirmed by demonstrating that intensive reducing of the LDL cholesterol below 1.8 mmol/L, does not change the gene expression of the markers involved in the inflammatory and migration process in both, monocytes and progenitor cells in the bone marrow (*42*). At the same time, the CCR2 expression remained unchanged on both cell types (*42*). Surface marker CCR2 is expressed on monocytes/macrophages and ECs. The interaction of CCR2 with its ligand, *i.e.* chemoattractant chemokine ligand 2 (CCL2), enables the adhesion and penetration of the monocytes/macrophages into the subendothelial space, allowing the formation of the foam cells (*20*). In the CCR2 knock out (-/-) mice, even in the presence of CCL2, the ability of the monocytes/macrophages to adhere to ECs and migrate into the subendothelial layer is significantly impaired (*44*).

Lipoprotein(a) is a complex plasma protein that consists of LDL cholesterol and apolipoprotein B-100 (apoB) linked to the plasminogen-like apolipoprotein(a) (apo(a)) *via* a disulphide bond. Lipoprotein(a) is suggested to possess several divergent functions. These include proatherosclerotic due to the similarity with LDL cholesterol, and prothrombotic effects due to similarity between apo(a) and plasminogen. On the other hand, the pro-inflammatory effects are mainly due to OxPLs activating monocytes and ECs (*45*). A substantial number of monocytes was shown to accumulate in the vessel wall in patients with increased Lp(a) values compared to those with normal values (*46*). These results suggest that increased values ​​of Lp(a) play an important role in the local inflammatory process in the vascular wall. However, there is no evidence from the clinical studies, as no drugs that specifically lower Lp(a) are available. Treatment with PCSK9 inhibitors lowers LDL cholesterol concentrations by 60% and Lp(a) concentrations by 20-30%, however it does not reduce the vessel wall inflammation in patients previously treated with statins (*47*). Treatment with specific drugs reduces Lp(a) concentrations by up to 90% and reduces the pro-inflammatory state of circulating monocytes (*48*). Lipoprotein(a) is the main carrier of OxPL, which is one of the most important activators of both, the monocytes and the ECs. Study by van der Valk *et al.* showed more notable inflammation in the vascular wall of the patients with increased Lp(a) values ​​in comparison with controls with normal Lp(a) values (*46*). They demonstrated that the uptake of 18F-fluorodeoxyglucose, which is an accurate indicator of local atherogenic inflammation, into the vascular wall is directly proportional to the concentration of Lp(a). At the same time, they showed that areas with greater inflammation coincide with an increased accumulation of the peripheral blood mononuclear cells. Interestingly, this was evident not only in the areas with atherosclerotic lesions, but also in the apparently unaffected vessel walls (*46*). Moreover, the monocytes of patients with increased Lp(a) values secrete larger amounts of the pro-inflammatory cytokines such as IL-1, IL-6 and TNF-α, but on the other hand, the secretion of the anti-inflammatory cytokines such as IL-10, is significantly reduced. The monocytes from patients with increased Lp(a) values have also an increased ability to migrate through the endothelial barrier. Van der Valk *et al.* also showed that Lp(a) containing OxPLs increases the inflammatory response of the monocytes from patients with normal Lp(a) values (*46*). The proinflammatory effects of apo(a) can be blocked by a specific antibody E06. Finally, the authors provide evidence on the necessity for OxPL mediated monocyte activation. The r-apo(a) that contains bound OxPL is capable of activating monocytes, whereas nearly identical but mutated r-apo(a) without the ability to bind OxPL, does not possess the monocyte activation properties. To summarize, these data indicate that the OxPLs carried by Lp(a), are obligatory danger signals in eliciting the prolonged potentiation of the monocyte response *in vitro* (*46*).

An increased concentration of Lp(a) is also associated with an increased number of specific subtypes of monocytes, in particular those with the pro-inflammatory role in the atherosclerosis process. In a cohort of 90 patients with stable coronary disease, Khrystiuk *et al.* showed that patients with increased Lp(a) values had a significantly higher proportion of IMs compared to patients with normal values (*49*). More importantly, the OxPLs/apoB ratio was increased in patients with increased Lp(a) values, underpinning the pro-inflammatory role of Lp(a). Additionally, in the group with increased Lp(a) values, the concentration of CRP and IL-6 was also higher. Similar to LDL cholesterol, oxidized form of Lp(a), *i.e.* OxLp(a) is considered to be even more atherogenic than its native form. Namely, OxLp(a) increases the synthesis of ROS through various signalling pathways and thus increases the permeability of ECs (*50*). In patients with type II diabetes, endothelial function was more impaired in those with elevated OxLp(a)/Lp(a) ratio (*51*). Not only functional but also morphological changes of the endothelium are associated with the concentration of OxLp(a) rather than with its native form, *i.e.* Lp(a) (*52*).

An increased concentration of Lp(a) does not show proatherogenic effects only on monocytes/macrophages, but also on ECs. These cells present the last barrier that monocytes/macrophages have to overcome to move into the subendothelial space. Lp(a) triggers pro-inflammatory response of ECs, which begin to release larger amounts of inflammatory cytokines, in particular IL-6 and IL-8. At the same time, the increased expression of the adhesion molecules such as ICAM-1, E-selectin and CCR2, strongly increases the transendothelial migration of monocytes (*53*). The apo(a) part, which also contains OxPLs, is responsible for these effects. The expression of the aforementioned cytokines and selectins increased in both, human umbilical vein ECs, as well as in coronary artery ECs under the influence of Lp(a) or apo(a) stimulation (*54*, *55*). Moreover, the increased release of the adhesion molecules from ECs was shown to be dependent on the Lp(a) concentration and the exposure time (*54*, *55*). Not only does the increased concentration of Lp(a) and in particular apo(a) trigger the expression of cytokines, selectins and ROS, it does cause changes in the cytoskeleton of the ECs as well. These changes lead to greater permeability of the ECs and thus facilitate the migration of the monocytes/macrophages into the subendothelial space (*56*). Apolipoprotein(a), through its strong lysine-binding site in KIV(10’), mediates the increased contraction of the ECs and permeability *via* a Rho/Rho kinase/MYPT1-dependent pathway (*56*).

## Monocytes and macrophages in the progression of atherosclerosis

After monocytes/macrophages penetrate into the subendothelial space, they accumulate lipoproteins and turn into foam cells, transforming functional changes of the endothelium into morphological. Foam cells are involved in the formation of the atherosclerotic plaque, its growth and, in case of its rupture, in the occurrence of an acute cardiovascular event. The most important risk factor for the formation of foam cells is an increased concentration of lipoproteins, in particular LDL cholesterol and Lp(a) (*57*, *58*). The formation of the foam cells results with interweaving of the three processes: lipid uptake, lipid efflux and cholesterol esterification. Increased accumulation of the lipoproteins and cholesterol esters in macrophages suggests that lipid uptake and cholesterol esterification dominate cholesterol efflux (*59*).

The most important pathway for cholesterol efflux is provided by the scavenger receptors (SRs), among which scavenger receptor A (SR-A) and CD36 stand out, as they contribute between 75 and 90% of the uptake of the modified lipoproteins (*60*). Cholesterol efflux from the foam cells occurs with diffusion and with the help of various transport systems. Under normal circumstances, diffusion is the major contributor to lipoprotein efflux, while in the case of the increased lipoprotein concentration, the main part of the lipoprotein efflux happens through SR class B type 1 (SR-B1), ATP binding cassette transporter A-1 (ABCA1) and ATP-binding cassette sub-family G member 1 (ABCG1) (*61*). Cholesterol from the foam cells can be transferred by HDL cholesterol or apolipoprotein A1 (apoA1), presenting the first step in reverse cholesterol transport and one of the possible mechanisms for the atheroprotective role of the HDL cholesterol (*62*). Esterification is the most important process for storing cholesterol in all cells, including in the foam cells that form the atherosclerotic lesions (*63*). Acyl-coenzyme A cholesterol acyltransferase (ACAT) is an enzyme, responsible for cholesterol esterification in the macrophages. Its inhibition in mice reduces foam cell formation and atherosclerosis progression, but has no effect on atherosclerosis progression in carotid and coronary arteries in patients (*64*). Similar to OxLp(a), OxLDL cholesterol is even more atherogenic than its native form. This was demonstrated by Ehara *et al.* who compared the concentration of OxLDL cholesterol in patients with acute coronary syndrome, unstable and stable angina pectoris and control group (*65*). They showed that the concentration of OxLDL cholesterol is related to the severity of the coronary atherosclerosis. This association was independent of other risk factors, including LDL cholesterol concentrations. More importantly, the concentration of OxLDL cholesterol in atherectomy specimens was significantly higher in patients with unstable angina pectoris compared to the patients with stable coronary disease. In order to prove the connection between the OxLDL cholesterol and the foam cells or macrophages, they also compared the surface area of ​​OxLDL positive macrophages between these groups of patients and showed it was significantly higher in patients with unstable angina pectoris. The majority of the OxLDL cholesterol is absorbed into macrophages *via* the lectin-like oxidized low-density lipoprotein receptor-1 (LOX-1). Low-density lipoprotein receptor-1 is present on the surface of macrophages, ECs and smooth muscle cells, suggesting the involvement of the OxLDL in all phases of the atherosclerotic process – from endothelial dysfunction to rupture of the atherosclerotic plaque and the resulting acute cardiovascular event (*66*). Furthermore, OxLDL cholesterol can trigger arterial thrombosis by activating platelets adhesion and reducing the fibrinolytic capacity of ECs (*67*). Arterial thrombosis is primarily associated with the rupture of the atherosclerotic plaque, and the release of large amounts of TF (*68*). Exposure of TF to blood initiates the extrinsic clotting cascade, and is considered to be a major regulator of coagulation (*69*).

Patients with FH, who in addition to extremely increased LDL cholesterol values, also have increased Lp(a) values, have a higher risk of an acute coronary event compared to FH patients with normal Lp(a) values (*70*). Increased values ​​of Lp(a) are associated with the composition of the atherosclerotic plaque. In patients with acute coronary syndrome requiring percutaneous coronary intervention optical coherence tomography of culprit lesion showed that fibrosus cap thickness was significantly smaller in those with increased Lp(a) concentrations compared to those with normal Lp(a) concentrations (*71*). In patients with symptomatic carotid atherosclerosis, increased Lp(a) concentration was associated with lipid-rich necrotic core independently of other risk factors, including LDL cholesterol concentration (*72*).

Long-term, *i.e.* at least 10 years, prospective research showed that the progression of both, carotid and coronary atherosclerosis is faster in patients with higher ​​than in patients with lower Lp(a) concentrations (*73*, *74*). In patients with higher Lp(a) concentrations, the lipid-rich necrotic core significantly increases and the fibrosus cap thickness decreases, which is associated with increased tendency for rupture and a higher probability of arterial thrombosis at the site of the atherosclerotic plaque rupture. The increase in lipid-rich necrotic core is most likely related to the pro-inflammatory properties of Lp(a).

Freshly isolated monocytes from patients with elevated Lp(a) concentrations ​​show three times higher transendothelial migration capacity compared to monocytes from control patients (*46*). At the same time, their monocytes possess significantly more scavenger receptors CD36 and SR-A on their surface, which also contributes to significantly higher uptake of the lipoproteins and faster growth of the foam cells. At the same time, these monocytes secrete larger quantities of the pro-inflammatory cytokines, for example IL-6, TNF-α and IL-1β, and significantly smaller amounts of the anti-inflammatory cytokine IL-10. This creates a vicious circle that contributes to a faster formation of more vulnerable atherosclerotic plaques. All these processes are directly related to higher Lp(a) concentrations (*46*). As already mentioned, the pro-inflammatory action of Lp(a) is largely due to OxPLs. Several studies involving more than 30,000 individuals, found that the concentration of Lp(a) is related to the OxPLs/apoB ratio and inversely depends on the size of the apo(a) isoform (*75*, *76*). The OxPLs/apoB ratio proved to be a predictive factor for future coronary events independent of other risk factors except of Lp(a) concentration (*77*). Oxidized phospholipids are rapidly transferred to Lp(a), and become predominantly associated with Lp(a) compared to other apoB-containing lipoproteins, despite the particle number of Lp(a) in plasma being generally much lower than that of the LDL cholesterol (*78*). This may also explain why Lp(a) is so much more atherogenic than the LDL cholesterol (*79*).

Upon rupture of an atherosclerotic plaque with many lipid-rich necrotic cores, a large amount of TF is released, which is stored in foam cells, monocytes/macrophages and smooth muscle cells. At the same time, a large amount of IL-6, IL-1 and TNF-α is released from a plaque, which further increases the production of TF in all the aforementioned cells (*80*).

Lipoprotein(a) participates in atherothrombosis through several mechanisms. As an atherogenic lipoprotein, Lp(a) interferes with platelet aggregation, as it can bind to platelet-activating factor acetyl hydrolase, which degrades and inactivates platelet-activating factor. This results in reduced platelet aggregation and activation. When plasminogen is activated to plasmin by either t-PA or u-PA action, the resulting enzyme cleaves several substrates, including fibrin, resulting in dissolution of the thrombi through fibrinolysis. The thrombogenic properties of Lp(a) might be due to the homology between apo(a) and plasminogen. Lipoprotein(a) competes with plasminogen for binding sites on ECs, which inhibits fibrinolysis and promotes intravascular thrombosis (*45*). However, there is no evidence for these speculations, as Mendelian randomization studies were not able to confirm the connection between Lp(a) and venous thromboembolism (*81*). Namely, it turned out that not the entire Lp(a), but the apo(a) alone, successfully inhibits the fibrinolytic process (*82*, *83*). However, this by no means excludes the role of Lp(a) in the development of the arterial thrombosis. It rather suggests the involvement of other mechanisms independent of the plasminogen activation. It was shown that Lp(a) inhibits TF pathway inhibitor and thus accelerates the coagulation process (*84*). At the same time, it was also proved that an increased concentration of Lp(a) stimulates the formation of PAI-1 in ECs and in this way affects the fibrinolytic activity (*85*).

## Clinical utility and limitations

Lowering LDL cholesterol with statins is a well-recognized and one of the most effective strategies for reducing cardiovascular events (*86*). However, at least part of the statin efficacy can be also attributed to their anti-inflammatory effect that is independent of their lipolytic action (*87*). Treatment with PCSK9 inhibitors in patients previously treated with statins did not further decrease the inflammatory parameters, however it substantially reduced the inflammation in the vessel wall (*88*). On the contrary, treatment with PCSK9 inhibitors significantly reduced the concentration of the inflammatory parameters in statin naïve patients (*89*). Treatment with PCSK9 inhibitors decreased Lp(a) concentration, however only 20-40%. The study was not designed to examine the impact of the decrease in Lp(a) on the inflammation in the vessel wall (*89*). However, it is difficult to estimate how much of the reduction in inflammation in the vascular wall is due to the lowering of LDL cholesterol, and how much is due to the lowering of Lp(a). Although the reduction in the incidence of recurrent cardiovascular events in studies with PCSK9 inhibitors has been shown to depend on both the reduction in LDL cholesterol and Lp(a) concentrations (*90*). Of course, we cannot precisely separate the contribution of both. Thus, we cannot precisely determine the contribution of both reductions in reducing inflammation in the vascular wall, which is not dependent on a decrease in markers of inflammation in the blood. Drugs that reduce Lp(a) by up to 90% are currently in the phase III of clinical trial (*91*). Since the inflammation in the vascular wall is associated with increased Lp(a) values, we can anticipate that this type of treatment will also have an additional impact on ameliorating the inflammatory process in the vascular wall in patients with increased Lp(a) values that present a high risk group for future coronary events. Furthermore, it is reasonable to expect that the novel treatment if started very early at the beginning of the development of the atherosclerotic process would be even more effective in preventing the occurrence of cardiovascular events.

Of course, our review article also has shortcomings. The most important is that we probably do not have data from randomized trials on the impact of reducing inflammation in the vascular wall on the incidence of cardiovascular events, and we probably will not have them, at least in the near future. Such results would require a randomized double-blind study that would simultaneously monitor the impact of reducing inflammation in the vascular wall and the incidence of cardiovascular events under the influence of the tested drug. In the case of patients with increased concentrations of both LDL cholesterol and Lp(a), this would be much more difficult to implement. Namely, we would need several groups that, in addition to placebo, would receive drugs that effectively lower only LDL cholesterol or Lp(a), and of course their combination.

## Conclusions

Atherosclerosis is no longer considered a passive process of deposition of lipids in the arterial vessel wall. It is an active process of interaction between lipoproteins, in particular the most atherogenic LDL cholesterol and Lp(a), and inflammatory cells, mainly monocytes/macrophages. The increased concentration of the atherogenic lipoproteins enables and accelerates the formation of the foam cells from monocytes/macrophages, which then release large quantities of inflammatory cytokines that stimulate the oxidation of the atherogenic lipoproteins. Oxidized phospholipids are even more atherogenic and contribute to the formation of foam cells and the secretion of the pro-inflammatory cytokines, thus creating a vicious circle. This vicious cycle can be interrupted by affecting the concentration of either OxLPs or pro-inflammatory cytokines, or preferably both.

## Data Availability

No data was generated during this study, so data sharing statement is not applicable to this article.
